# MRI-Based Classification Models in Prediction of Mild Cognitive Impairment and Dementia in Late-Life Depression

**DOI:** 10.3389/fnagi.2017.00013

**Published:** 2017-02-02

**Authors:** Aleksandra K. Lebedeva, Eric Westman, Tom Borza, Mona K. Beyer, Knut Engedal, Dag Aarsland, Geir Selbaek, Asta K. Haberg

**Affiliations:** ^1^Department of Neurobiology, Care Sciences and Society, Karolinska InstitutetStockholm, Sweden; ^2^Centre for Old Age Psychiatric Research, Innlandet Hospital TrustBrumunddal, Norway; ^3^Institute of Clinical Medicine, Faculty of Medicine, University of OsloOslo, Norway; ^4^Department of Radiology and Nuclear Medicine, Oslo University Hospital, RikshospitaletOslo, Norway; ^5^Department of Geriatric Medicine, Oslo University HospitalTønsberg, Norway; ^6^Oslo and Norwegian National Advisory Unit for Aging and HealthTønsberg, Norway; ^7^Department of Old Age Psychiatry, Institute of Psychiatry, Psychology and Neuroscience, King’s College LondonLondon, UK; ^8^Center for Age-Related Medicine, Stavanger University HospitalStavanger, Norway; ^9^Department of Neuroscience, Norwegian University of Science and TechnologyTrondheim, Norway; ^10^Department of Radiology and Nuclear Medicine, St. Olav’s University HospitalTrondheim, Norway

**Keywords:** depression, MCI, Alzheimer’s disease, Freesurfer, neurodegeneration, hypothalamus, ventral diencephalon, depressive episodes

## Abstract

**Objective:** Late-life depression (LLD) is associated with development of different types of dementia. Identification of LLD patients, who will develop cognitive decline, i.e., the early stage of dementia would help to implement interventions earlier. The purpose of this study was to assess whether structural brain magnetic resonance imaging (MRI) in LLD patients can predict mild cognitive impairment (MCI) or dementia 1 year prior to the diagnosis.

**Methods:** LLD patients underwent brain MRI at baseline and repeated clinical assessment after 1-year. Structural brain measurements were obtained using Freesurfer software (v. 5.1) from the T1W brain MRI images. MRI-based Random Forest classifier was used to discriminate between LLD who developed MCI or dementia after 1-year follow-up and cognitively stable LLD. Additionally, a previously established Random Forest model trained on 185 patients with Alzheimer’s disease (AD) vs. 225 cognitively normal elderly from the Alzheimer’s disease Neuroimaging Initiative was tested on the LLD data set (ADNI model).

**Results:** MCI and dementia diagnoses were predicted in LLD patients with 76%/68%/84% accuracy/sensitivity/specificity. Adding the baseline Mini-Mental State Examination (MMSE) scores to the models improved accuracy/sensitivity/specificity to 81%/75%/86%. The best model predicted MCI status alone using MRI and baseline MMSE scores with accuracy/sensitivity/specificity of 89%/85%/90%. The most important region for all the models was right ventral diencephalon, including hypothalamus. Its volume correlated negatively with the number of depressive episodes. ADNI model trained on AD vs. Controls using SV could predict MCI-DEM patients with 67% accuracy.

**Conclusion:** LDD patients developing MCI and dementia can be discriminated from LLD patients remaining cognitively stable with good accuracy based on baseline structural MRI alone. Baseline MMSE score improves prediction accuracy. Ventral diencephalon, including the hypothalamus might play an important role in preservation of cognitive functions in LLD.

## Introduction

Depression is associated with accelerated brain aging (Simon et al., 2006; McKinney and Sibille, 2013; Koutsouleris et al., 2014), and is a risk factor for different types of dementia (Aziz and Steffens, 2013; Diniz et al., 2013; Byers and Yaffe, 2014; Cooper et al., 2015; Mourao et al., 2015).

Unfortunately, early identification of predementia states in people with LLD is challenging as reduced cognitive scores can be confounded by the depressive state. Thus depression is an exclusion criterion for some definitions of mild cognitive impairment (MCI; Steffens et al., 2006). It is challenging to predict whether cognitive impairment identified during a depressive episode in a LLD patient will improve after treatment of depression or will progress to dementia. However, it is known that among seniors with MCI identified during a depressive episode only 17% experienced cognitive improvement after 2 years of follow-up (Steffens et al., 2009). Early identification of those LLD patients with increased risk of progressive cognitive decline could allow for more targeted clinical actions, for instance choice of antidepressant (du Jardin et al., 2016; García-Fuster and García-Sevilla, 2016) or other interventions, (Ngandu et al., 2015) and possibly neuroprotective drugs in the future.

Biomarkers known from Alzheimer’s disease (AD) research may be used to identify a neurodegenerative process and/or increased risk of developing dementia in LLD patients in particular during a depressive episode when neurocognitive functions may not be reliably assessed. The biomarkers include amyloid-beta positron emission tomography (PET) brain imaging, magnetic resonance imaging (MRI) based cortical and subcortical structural measurements, cerebrospinal fluid tau, and amyloid-beta levels (American Psychiatric Association, 2013; Dickerson and Wolk, 2013). However, it is still not known if these biomarkers can be utilized to identify or predict future cognitive impairment (MCI or dementia status) in LLD.

The aims of this study were: (1) To assess whether structural T1 weighted (T1W) 3D brain MRI obtained during the depressive episode in LLD patients can discriminate LLD who were diagnosed with MCI or dementia after 1-year follow-up from cognitively stable LLD using Random Forest classifier. (2) To identify which regions are affected in LLD with subsequent cognitive impairment. (3) To evaluate the feasibility of using biomarkers derived from AD research, by implementing a classifier trained on structural 3D brain MRI from the Alzheimer’s Disease Neuroimaging Initiative (ADNI) sample.

## Materials and Methods

### Cohorts

**FIGURE 1:**
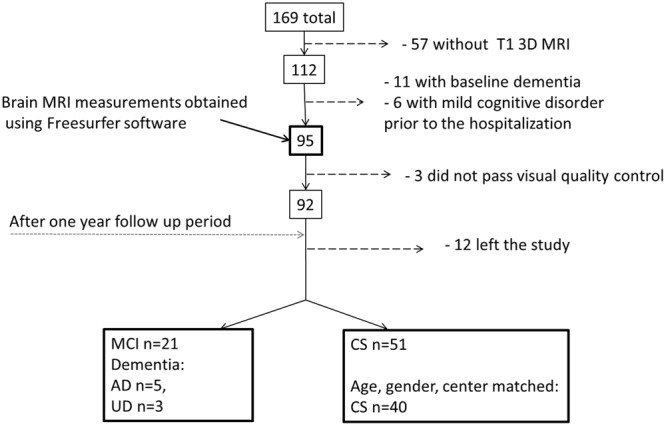
**Flow chart describing patient selection and attrition from admission to 1-year follow-up assessment.** AD, Alzheimer’s disease; UD, Unspecified dementia; MCI, mild cognitive impairment; CS, cognitively stable control group; mild cognitive disorder – F 06.7 (ICD-10).

Two cohorts were used in this study:

(1) PRODE (Prognosis of depression in the elderly) – was used as the main cohort (Borza et al., 2015). The PRODE study is a prospective multicenter study including 169 patients ≥60 years referred to treatment for depression at nine centers of geriatric psychiatry in Norway (Borza et al., 2015). Exclusion criteria were life threatening diseases and severe aphasia (as it would reduce the validity of the neuropsychological assessment). Clinical and neuropsychological data were collected using standardized clinical, psychiatric, and neuropsychological assessment (see below). Of the 169 patients, 126 underwent brain MRI examination at inclusion (Lebedeva et al., 2015) T1W 3D brain MRI images were available for 112 patients (**Figure 1**).
(2) ADNI – An established classification model based on RF trained to discriminate between patients with AD (*n* = 185) and healthy controls (HC, *n* = 225) using Freesurfer (v. 5.1) measurements from T1W 3D brain MRI was also used on the PRODE dataset. The training data was obtained from the ADNI database^1^. The ADNI was launched in 2003 as a public-private partnership, led by Principal Investigator Michael W. Weiner, MD. The primary goal of ADNI has been to test whether serial MRI, PET, other biological markers, and clinical and neuropsychological assessment can be combined to measure the progression of MCI and early AD. AD patients met the NINCDS/ADRDA criteria for probable AD. The T1W 3D MRI-Freesurfer based model had AD vs. HC accuracy of 90% for the testing dataset and predicted conversion in additional sample of MCI (*n* = 165) to AD with accuracy 79%. The most important regions for the discrimination were: left and right hippocampi, left amygdala, left and right entorhinal cortices, and left inferior temporal cortex (Lebedev et al., 2014). We hypothesized that this model could be used to predict MCI/AD conversion in the PRODE cohort.

### Clinical and Neuropsychological Assessment

Psychiatric evaluation and neuropsychological assessment were performed three times: at admission to the department of geriatric psychiatry (baseline) and discharge, and after 1-year. At each participating center trained clinicians performed the psychiatric and neuropsychological assessments using a harmonized procedure and the same protocol (Borza et al., 2015).

Clinical assessments included: demographic information, history of depression or other psychiatric condition, family history of psychiatric problems, current psychiatric problems, and treatment(s). Patients were diagnosed based on the criteria of the 10th Revision of the International Classification of Diseases and Health Related Problems (ICD-10; World Health Organization [WHO], 1993). Cognition was measured with the Mini Mental Status Examination, MMSE (Folstein et al., 1975) and Informant Questionnaire on Cognitive Decline in the Elderly (IQCODE-16; Jorm and Jacomb, 1989). At the 1-year follow-up assessment patients were also diagnosed using Winblad’s criteria for MCI (Winblad et al., 2004).

### Patient Selection

Patients without dementia diagnoses according to ICD-10 criteria at inclusion and a T1W 3D/structural brain MRI were included in this study (*n* = 95, **Figure 1**). Not all the patients could be included in the final analyses for a variety of reasons, as specified in **Figure 1**. Based on the 1-year follow-up diagnosis the patients were divided into one mild cognitive impairment-dementia (MCI-DEM) group including those diagnosed with either MCI (*n* = 21; Winblad et al., 2004) or dementia (*n* = 8, including: Alzheimer’s disease *n* = 5, unspecified dementia *n* = 3) [World Health Organization (WHO)]. A matched cognitively stable (CS) group was selected from the remaining 51 patient based on age and gender (*n* = 40) and controlling for the balance in number of CS and MCI at each center of inclusion (**Figure 1**).

### Ethics Statement

The participating patients and caregivers were given oral and written information, and they subsequently gave their written consent to participate. The PRODE study was approved by Regional Committee of Medical Research Ethics and Privacy and Data Protection Officer at Oslo University Hospital (approval number 2009/1774).

### MRI

#### MRI Acquisition

All scans were optimized and harmonized across centers based on American College of Radiology (ACR) phantom and a healthy volunteer. This study is based on T1 3D brain MRI obtained with sagittal volumetric magnetization-prepared rapid gradient echo (3D MP-RAGE) images using the ADNI T1W volume protocol (Jack et al., 2008) in six PRODE centers with 1.5T and 3T MRI scanners (see Supplementary Table S1). Inter-site reliability for the six centers was estimated using the intra-class correlation coefficient (ICC) using two-way random effect model with absolute agreement (McGraw and Wong, 1996). ICC was estimated for the average CTH and total volume of subcortical structures (TSV) obtained from the Freesurfer parcellation and segmentation (see below). Reliability test was performed using SPSS software v. 22. Assessment showed acceptable results (Fleiss, 1986). ICC (CTH) = 0.88 and ICC (TSV) = 0.98. It has been shown that Freesurfer output measurements are consistent between 1.5T and 3T MRI (Han et al., 2006; Pfefferbaum et al., 2012), thus the data across the field strengths was merged. CS group was matched on number of participants having 1.5 and 3T scans in addition to the other criteria.

#### Image Processing

**FIGURE 2:**
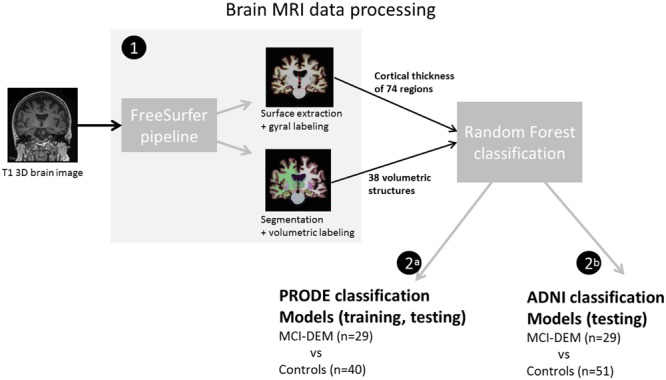
**Diagram of the brain magnetic resonance imaging (MRI) data processing (1) T1W 3D MRI brain images were analyzed in Freesurfer software. Resulting measurements where used as input for the random forest classifications. (2a)** In the prognosis of depression in the elderly (PRODE) models, PRODE cohort was used as training and testing datasets. **(2b)** The Alzheimer’s Disease Neuroimaging Initiative (ADNI) models were trained on the ADNI data (Alzheimer’s patients vs. healthy elderly). PRODE cohort was used as testing dataset.

Cortical thickness and SV were used as main outcomes. The T1W 3D MRI brain images were processed using Freesurfer (v. 5.1), where regional cortical thicknesses and volumetric measures were estimated. The software is well documented and available for download online^2^. The processing steps included cortical reconstruction and segmentation of gray matter volumetric structures. This was followed by parcellation of the cerebral cortex into units based on gyral and sulcal structure (Destrieux et al., 2010). One hundred forty eight CTH measures (74 from each hemisphere) and 54 regional volumes were generated. All output was visually inspected for segmentation and parcellation quality before the analysis. Volumes of left and right white matter hypointensities, optic chiasm, right and left vessel, and left and right choroid plexus were excluded from further analysis. List of measurements included in the analysis is provided in Supplementary Table S2. The accuracy of the CTH and hippocampal volumes measurements derived by this technique has been validated by histological and manual measurements (Rosas et al., 2002; Sánchez-Benavides et al., 2010). All volumetric structures were normalized by the subject’s intracranial volume using the residual approach (Jack et al., 1989). Brain structures in the left and right hemisphere may have different degree of atrophy, thus CTH and SV of the left and right hemisphere were treated separately (Dolcos et al., 2002). Workflow is represented in **Figure 2**.

### Statistical Analysis

R programming language (R Core Team, 2016), version 3.3.0, was used to compare demographic and clinical data, and to create random-forest algorithm (RF) classification models. For group comparisons of demographic and clinical variables, the chi-squared test was used for categorical variables, and *t*-test or Mann–Whitney where appropriate for continuous variables. RF classification models were established using R packages “random forest” (Liaw and Wiener, 2002) and “caret” (Kuhn et al., 2016), ROC-curve and area under the ROC-curve (AUC) were estimated and plotted using “pROC” package (Robin et al., 2011). Regions important for the classification were correlated with clinical and demographic variables.

### Random Forest Algorithm and Performance Assessment

Random forest method allows performing supervised classifications based on an ensemble of classification trees (Breiman, 2001). RF selects a bootstrapped subset of all observations – about 66% per tree and random subset of all predictors/features (here: CTH, SV) at each node of the tree. RF uses the majority vote of its trees terminal nodes to predict the class label of a new observation. Each tree casts a unit vote for the class. Thus, high numbers of decision trees are expected to increase reliability of the results. For each predictor a Gini index is estimated at each node. Overall importance of a predictor for the model is based on the summation of the decreases in the Gini index at each node (Breiman, 2001). The remaining 33% of the data, i.e., out-of-bag (OOB) data, is used to measure the RF performance. The classification error of the OOB observations is referred to as OOB error (Breiman, 1996). Kappa is another measure of performance demonstrating how close the RF classifications were to the actual classes, controlling for the accuracy of a random classifier as measured by the expected accuracy. Kappa is suggested to provide more reliable information regarding the classifier performance than actual accuracy in case of different class distribution in the dataset (Fleiss, 1971).

#### RF Procedure

Random forest method algorithm was used to discriminate between the MCI-DEM or MCI and CS groups at 1-year follow-up based on the CTH and SV measures separately and combined. In addition, demographic and clinical information was added to the models to test if performance could be further improved. Only the clinical information obtained at inclusion was used to assess if MCI-DEM statuses 1-year later could be predicted based on the earliest available clinical data and in the depressive state.

#### PRODE Cohort Models

##### PRODE models

Five thousand decision trees were used in the RF classification models. RF models were trained to discriminate between MCI-DEM (*n* = 29) or MCI alone (*n* = 21) and corresponding CS patients. When discriminating between MCI vs. CS, the number of CS was reduced to 30 (from 40) matched on age and gender and scanner field strength, in order to keep balance in group class distribution. AUC, sensitivity/specificity, overall accuracy, and kappa, were used to assess performance of the models. Confidence intervals (CI) were estimated using bootstrapping (*n* = 100). The most relevant structures for prediction of MCI-DEM or MCI were correlated with clinical variables related to depression in order to detect regions involved in both pathological processes.

##### ADNI cohort models

The previously established ADNI model (Lebedev et al., 2014) was implemented in the PRODE dataset to evaluate its ability to discriminate between MCI-DEM (*n* = 29) and CS group (*n* = 51). However, we did not expect the ADNI model to outperform the PRODE models because it was trained on a data derived from AD patients versus HC, not LLD and not converting to MCI.

## Results

### Demographics

Clinical and demographic variables in MCI-DEM and CS groups are provided in **Table 1**. MCI-DEM group had significantly lower MMSE scores at all three time points compared with CS group. Other variables did not differ between the groups. Similarly, the MCI group alone did not differ from the matched CS patients in terms of age (*p* = 0.21) or gender (*p* = 0.92). Notably, the standard deviation of the MMSE scores increased in the MCI/dementia group between assessments “at discharge” and “follow-up,” reflecting increased variance in their cognitive statuses.

**Table 1:** Demographic and clinical characteristics of the MCI-DEM and cognitively stable (CS) groups.

PRODE data set	MCI-DEM	CS	Test statistics	*p*-value^∗^
*N*	29	40	–	–
Age – mean (SD)	78.1 (7,3)	76,4 (5,8)	*t* = -1.5	0.31
Sex – n women (%)	22 (75%)	29 (72%)	χ^2^ = 0.03	0.85
Education (total years) – mean (*SD*)	8.9 (2.79)	10.1 (2.61)	*t* = -1.8	0,07
Number of depressive episodes – (range)	2.5 (0–4)	1.5 (0–4)	*z* = -1.06	0.28
Age at depression onset – median (range)	29.7 (15–85)	35.0 (16–88)	*z* = -1.02	0.3
MADRS-1 – median (range)	26 (15–52)	27 (0–48)	*z* = -0.66	0.5
MADRS-2	9 (0–31)	7 (0–30)	*z* = -0.44	0.65
MADRS-3	8 (0–35)	4.5 (0–30)	*z* = -0.43	0.66
MMSE-1 – mean (*SD*)	24.6 (3.0)	26.4 (1,8)	*t* = -2.71	0.005
MMSE-2	25 (2.8)	27 (1.9)	*t* = -5.42	0.009
MMSE-3	23.8 (5.6)	28 (1.7)	*t* = -5.90	<0.001

*MCI-DEM – LLD patients who received diagnosis of mild cognitive impairment or dementia at 1-year follow-up assessment. CS – LLD patients who remain cognitively stable at 1-year follow-up assessment.*

*^∗^*t*-test, Mann–Whitney *U* test or chi-square test as appropriate.*

*MADRS, Montgomery–Asberg Depression Rating Scale; MMSE, Mini Mental State Examination.*

*MADRS/MMSE – 1 - at inclusion, 2- at discharge, 3- after 1 year follow-up period.*

### Prediction of MCI and Dementia in LLD

#### PRODE (MCI-DEM vs. CS Group) Models

The model using SV+CTH as input had the best performance in discrimination between MCI-DEM and CS groups. The model using only SV as input had the best performance in discrimination between MCI (excluding eight dementia patients) and CS groups. Details are provided in **Table 2**. The model using only CTH as input for the MCI vs. CS discrimination had 67% accuracy and sensitivity < 50%. MMSE score at inclusion improved the models performance. Adding age, gender, or education to the model did not affect the results. When excluding patients with MADRS < 7 and MMSE < 26 at discharge (*n* = 4), accuracy changed slightly from 76 to 74% using SV as input.

**Table 2:** Performance of the random forest classification models based on baseline structural magnetic resonance imaging (MRI) measurements with and without Mini-Mental State Examination scores.

Model	AUC (95% CI)	Sens/spec(accuracy)	kappa
**PRODE**	**Training set: MCI-DEM (*n* = 29) vs. CS group (*n* = 40)**^∗^		
SV	0.802 (70.4%–90.0%)	68/84 (74.6)	0.46
CTH+SV	0.758 (64.5%–87.0%)	55/86 (76.0)	0.47
CTH+SV+MMSE1	0.867 (77.5%–95.9%)	75/86 (81.3)	0.61
SV+MMSE1	0.892 (75.1%–96.9%)	75/81 (81.3)	0.60

**PRODE**	**Training set: MCI (*n* = 21) vs. CS group (*n* = 30)**^∗^		
SV	0.773 (64.3%–90.3%)	65/86 (76.4)	0.50
CTH+SV	0.678 (51.78%–83.8%)	52/80 (70.5)	0.44
CTH+SV+MMSE1	0.867 (75.1%–98.2%)	76/90 (86.2)	0.71
SV+MMSE1	0.905 (81.4%–99.6%)	85/90 (90.1)	0.79

**ADNI**	**Training set: ADNI AD vs. ADNI Controls**		
	**Testing set: PRODE MCI-DEM (*n* = 29) vs. PRODE CS group (*n* = 51)**^∗∗^		
SV	0.737 (62.6%–84.7%)	65.5/68.6 (67)	0.40
CTH+SV	0.623 (50.1%–74.4%)	48/62 (57.5)	0.10

*PRODE model – trained to discriminate between LLD who developed MCI or dementia and cognitively stable LLD.*

*ADNI model – trained to discriminate between non-depressed patients with Alzheimer’s disease and cognitively normal non-depressed elderly.*

*CTH, cortical thickness; SV, subcortical volumes; MC, mean curvature; OOB, out-of-bag; AUC, area under the ROC-curve; MMSE1, Mini–Mental State Examination scores at admission; Sens/Spec, Sensitivity/specificity; NA, not applicable.*

*^∗^ Accuracy estimated for OOB set.*

*^∗∗^Accuracy Estimated for the testing dataset.*

#### Brain Measurements Used in Prediction

The most relevant measurements for the models were right ventral diencephalon (R-VD, mean decrease in Gini index = 8.26), middle anterior corpus callosum (mid-anterior CC, mean decrease in Gini index = 2.06) and right hippocampus (R-HC mean decrease in Gini index = 1.47). For all the PRODE models (**Table 2**), the same structures (R-VD, mid-anterior CC, and R-HC) were the most important. When excluding participants from each center, the same structures remained the most important.

#### Relationship between Ventral Diencephalon and Clinical and Demographic Measures

Right ventral diencephalon, mid-anterior CC, and R-HC volumes were correlated with the number of depressive episodes (adjusting for age, gender, and MMSE). The number of depressive episodes had significant inverse association with R-VD (*p* = 0.02) and mid-anterior CC (*p* = 0.04) volumes. Next, total ventricular volume was added as a covariate to assess if structural changes co-occur with ventricle expansion as an indirect measure of a degenerative nature of the observed structural changes. Adjusting for total ventricular volume negated the effect of number of depressive episodes on R-VD volume (*p* = 0.12) and mid-anterior CC volume (*p* = 0.08). No association was found between the number of depressive episodes and R-HC volume. MMSE score was associated with R-HC volume (*p* = 0.03), but not with R-VD volume (*p* = 0.16) or mid-anterior CC volume (*p* = 0.18).

#### ADNI (MCI-DEM vs. CS Group) Model

The ADNI model trained on SV demonstrated better performance on separating MCI-DEM vs. CS in the PRODE dataset (accuracy = 67.0%) than the ADNI model trained on CTH+SV (accuracy = 57.5%; **Table 2**).

## Discussion

In this study, we have demonstrated that LLD patients who were diagnosed with MCI or dementia 1 year later can be discriminated from cognitively stable LLD patients using structural brain measurements with 76% accuracy. The best model predicted MCI status alone using SV and MMSE scores with accuracy/sensitivity/specificity of 89%/85%/90%.

To our knowledge this study is the first to build classification models based on structural MRI measures to predict development of MCI or dementia in LLD patients, and to assess performance of a model trained for AD-HC discrimination (ADNI model) on a LLD dataset (PRODE cohort). We used all brain parenchyma volumes derived from the Freesurfer analysis, and showed that volumes of R-VD, mid-anterior CC, and R-HC were the most important for discrimination between MCI-DEM and CS group.

We have found only one study using a classification approach and MRI data to predict MCI diagnosis, but in non-depressed elderly and based on arterial spin labeling. This study reported that perfusion in a region of interest in the posterior cingular cortex could distinguish those developing MCI from a CS group with an AUC of 66% (Xekardaki et al., 2015).

### Brain Regions Important for Prediction of MCI and Dementia Diagnosis

The most important regions for predicting a diagnosis of MCI or dementia after 1-year follow-up were volumes of R-VD, mid-anterior CC, and R-HC, respectively.

Right ventral diencephalon was demonstrated to be the most relevant structure. The ventral diencephalon in Freesurfer includes several structures: hypothalamus with mammillary body, subthalamic, lateral geniculate, medial geniculate and red nuclei, substantia nigra and surrounding white matter. Some of these structures, i.e., substantia nigra and red nuclei, are not locate to the diencephalon but mesencephalon according to standard anatomical nomenclature. The hypothalamus is known to be strongly involved in depression. As a part of hypothalamic–pituitary–adrenal (HPA) axis, it is crucial for emotional behavior and stress response. There are numerous studies showing dysregulation of the HPA axis in depression, but also in aging and neurodegeneration (Sapolsky et al., 1986; Sapolsky, 2000; Varghese and Brown, 2001; Du and Pang, 2015). It has also been proposed that HPA-axis dysfunction is central to the development of AD (Ishii and Iadecola, 2015). Indeed, hypothalamic dysfunction can explain the overlap in symptoms between depression and AD (mood, appetite, sleep, memory, autonomic). Consistent with our finding, several previous imaging studies have shown structural and functional abnormalities in the hypothalamus in MCI, preclinical AD, and AD compared with control groups (Callen et al., 2001; Nestor et al., 2003; Cross et al., 2013; Brueggen et al., 2015). For instance, Hall et al. (2008) have demonstrated reduced basal forebrain and hypothalamus volumes in preclinical AD, and interestingly the combination of reduced forebrain and hippocampal volumes was associated with more rapid cognitive decline. However, to the best of our knowledge the current study is the first study to assess the role of the entire ventral diencephalon in the context of neurodegeneration. There are two previous studies examining the ventral diencephalon (segmented in Freesurfer) in relation to mood disorders. The first showed that bilateral ventral diencephalon volume obtained from Freesurfer was one of three top-ranked endophenotypes of major depressive disorder in an analysis of a high-dimensional set of over 11,000 traits (Glahn et al., 2012). The other study demonstrated that volume of the ventral diencephalon discriminated patients with major depressive disorder from those with bipolar depression as well as controls (Sacchet et al., 2015). Taken together, these findings suggest that neurodegeneration in ventral diencephalon including the hypothalamus might be a link between depression and cognitive impairment.

After R-VD, CC (mid-anterior CC) and hippocampal (R-HC) volumes were the most relevant structures for predicting MCI-DEM. HPA-axis dysregulation is associated with elevated cortisol levels (O’Brien, 1996; Du and Pang, 2015) which is hypothesized to cause reduced CC and hippocampal volumes due to neurotoxic effects (Bao et al., 2008; Liu et al., 2016). The importance of CC volume for predicting MCI-DEM suggests presence of inter-hemispheric disconnection already in the early stages of the neurodegenerative process. Previously, it has been shown that anterior, middle and posterior portions of the CC have less volume in AD compared to controls, but only the middle part was smaller in amnestic MCI compared with controls (Qiu et al., 2016). Moreover, decreased volume of mid-anterior portion of CC has been linked to the memory loss in MCI and AD (Qiu et al., 2016). The present findings support a connection between R-VD, R-HC, and CC pathology in the development of MCI and dementia in LLD, which may be linked to HPA-axis dysregulation. A previous study examining only CC volume reported that structural changes in CC predicted MCI-to-AD conversion after 2.5 years on average (Lee et al., 2016), similar to the current study where a whole brain approach was used and conversion was to MCI/dementia. Hippocampal abnormalities are one of the most replicable findings in both depression and AD (Kempton et al., 2011; Sabuncu et al., 2011). Hippocampus has large bidirectional connection with the mammillary bodies of the hypothalamus which also might explain the concordant changes in these two structures. The limbic-diencephalic pathways, including the mammillothalamic tract and the mammillary bodies *per se* are crucial for episodic memory (Vann and Nelson, 2015; Aggleton et al., 2016). Hypometabolism in the mammillary bodies has been shown in both MCI and AD (Nestor et al., 2003). Limbic-diencephalic pathway dysregulation has been shown in the earliest stages of AD (Acosta-Cabronero and Nestor, 2014). Indeed, limbic regions, which are crucial for emotion processing, are also crucial for episodic memory. Thus, abnormal limbic-diencephalic interaction may be a core feature of in MCI-DEM development in LDD.

Of note, only brain structures from the right hemisphere were important for the classifications. This supports the right hemi-aging model, proposing that the right hemisphere shows greater age-related decline than the left hemisphere (Dolcos et al., 2002). However, this model is based on behavioral data rather than neuroimaging and the evidence has been controversial (Daselaar and Cabeza, 2005).

Interestingly, the number of depressive episodes had significant inverse association with the R-VD and mid-anterior CC volumes. Previously, it was reported that the number of depressive episodes is associated with reduced volume of the dentate gyrus in patients with major depressive disorder (Treadway et al., 2015). It is not known whether reductions of R-VD and mid-anterior CC are developmental phenomena, which leads to increase of the number of depressive episodes or degenerative consequences of a larger number of depressive episodes. However, including total ventricular volume to the regression model negated the effect of the number of depressive episodes on the R-VD volume and reduced on mid-anterior CC, providing indirect evidence for the degenerative nature of R-VD and mid-anterior CC reduction. Future studies using longitudinal imaging data could uncover the causality.

### SV+CTH+MMSE Models

MCI-DEM had significantly lower MMSE scores at all three time points compared to the CS group. Regardless of whether cognitive assessment in depressed patients is confounded by depression *per se*, including MMSE scores at admission improved the model’s prediction of MCI and dementia. Along with a recent study of Heser et al. (2016), the present results suggest that even during the depressive episode cognitive impairment require clinical attention as a possible sign of incipient dementia. Given the highly significant difference in MMSE scores at all three time points between the MCI/DEM and CS group, the probability that there was a true difference in MMSE scores between MCI-DEM and CS groups at all time points is very high, suggesting that MMSE in an appropriate additional predictor of MCI/AD even in LLD groups.

There was no correlation between MMSE scores R-VD and mid-anterior CC volumes suggesting that MMSE scores did not bias classification results. The absence of correlation between MMSE and the two main predictors suggested that MMSE improved classification models performance by explaining additional factor variance.

### Predicting MCI-DEM vs. MCI

The SV model trained on MCI vs. CS demonstrated better performance than the model trained on the mixed sample of MCI-DEM dementia vs. CS. The reasons might be a more heterogeneous pattern of structural brain changes across dementias in the MCI/DEM combined group and presence of larger variability in the stages of pathological process. On the other hand, the CTH+SV model performed better on the mixed MCI+DEM group compared with MCI only. One explanation might be that CTH get altered in the later stages and/or more severe cases of cognitive impairment in LLD and that relevance of the structural brain measurement as a biomarker therefore depends on the stage of the disease.

### Predicting MCI-DEM Conversion in LLD Using the ADNI Model

MCI-DEM status was discriminated from the CS group with 67% accuracy using the ADNI model. In other words, patients in the MCI-DEM group were more likely to be classified as cases than those in CS group based on their baseline brain MRI.

Interestingly, the ADNI model trained only on SV had much higher accuracy compared with the one trained on CTH+SV. Taking into account that the most relevant structures for MCI-CS discrimination (PRODE models) were subcortical structures and that the model combining SV with CTH had higher accuracy in MCI-DEM than in MCI alone, the present results may suggest that in LLD patients, who will develop MCI or dementia, neurodegeneration appears to start in subcortical structures and spread up to the neocortex in later stages. In agreement with our findings it has been shown that atrophy in basal forebrain and hypothalamus but not neocortex, precedes clinical symptoms of AD by 4.5–5 years (Brueggen et al., 2015). Several earlier studies have shown hypometabolism restricted to hippocampus and parahippocampal gyrus in MCI, whereas AD had additional temporal neocortical hypometabolism (De Santi et al., 2001; Nestor et al., 2003). Taken together, the current results suggest that classification models for prediction of MCI/preclinical AD should focus on subcortical structures rather than the neocortex.

### Limitations

One of the limitations of the present study is that the time interval between MRI assessment and MCI diagnosis was relatively short, thus it is possible that some CS patients could be diagnosed with MCI/dementia at a later time point. Excluding LLD participants with the lowest MADRS and MMSE scores at discharge (*n* = 4) did not alter the results notably (no substantial drop in accuracy) which indicate that the possibility that some patients could receive MCI diagnosis earlier did not bias the results strongly. Another issue is that MMSE might not be sensitive enough to detect MCI. In any case, there are no cognitive screening instruments validated for use in a depressed elderly population according to our knowledge. All the models including only brain measurements as predictors had very good specificity and sufficient sensitivity. One of the reasons for this might be the relatively small sample size. In future studies classification models should be trained on larger samples after longer follow-up periods. Inclusion of MRI scans obtained from scanners with different magnetic field strengths might be considered a limitation; however, it was shown that Freesurfer output measurements are consistent between 1.5T and 3T MRI, which was confirmed in our reliability analysis. We have shown that ICC is high across the centers. We also balanced the number of participants from each center and verified the results excluding each center, in order to assess reliability of the results which were consistent.

### Possible Practical Implementation

Our results suggest that LLD with smaller volumes of the R-VD, mid-anterior CC, and R-HC and lower MMSE scores at inclusion have a higher probability of receiving a diagnosis of MCI or dementia the following year. These measurements can provide clinicians with novel evidence for an expected trajectory of cognitive functioning in LLD and help to define a target group for interventions against cognitive decline (Bredesen, 2014).

Future mechanistic studies should verify processes underlying diencephalic neurodegeneration in LLD patients.

## Author Contributions

AL: Study design, data analyses, interpretation of the results, manuscript writing. EW and DA: Study design, interpretation of the results. TB and KE: Study design, data collection, interpretation of the results. MB: Data collection, interpretation of the results. GS and AH: Data collection, study design, interpretation of the results. All authors participated in manuscript revision and final approval.

### ADNI

Data collection and sharing for this project was funded by the Alzheimer’s Disease Neuroimaging Initiative (ADNI) (National Institutes of Health Grant U01 AG024904) and DOD ADNI (Department of Defense award number W81XWH-12-2-0012). ADNI is funded by the National Institute on Aging, the National Institute of Biomedical Imaging and Bioengineering, and through generous contributions from the following: AbbVie, Alzheimer’s Association; Alzheimer’s Drug Discovery Foundation; Araclon Biotech; BioClinica, Inc.; Biogen; Bristol-Myers Squibb Company; CereSpir, Inc.; Cogstate; Eisai Inc.; Elan Pharmaceuticals, Inc.; Eli Lilly and Company; EuroImmun; F. Hoffmann-La Roche Ltd. and its affiliated company Genentech, Inc.; Fujirebio; GE Healthcare; IXICO Ltd.; Janssen Alzheimer Immunotherapy Research & Development, LLC.; Johnson & Johnson Pharmaceutical Research & Development LLC.; Lumosity; Lundbeck; Merck & Co., Inc.; Meso Scale Diagnostics, LLC.; NeuroRx Research; Neurotrack Technologies; Novartis Pharmaceuticals Corporation; Pfizer Inc.; Piramal Imaging; Servier; Takeda Pharmaceutical Company; and Transition Therapeutics. The Canadian Institutes of Health Research is providing funds to support ADNI clinical sites in Canada. Private sector contributions are facilitated by the Foundation for the National Institutes of Health (www.fnih.org). The grantee organization is the Northern California Institute for Research and Education, and the study is coordinated by the Alzheimer’s Therapeutic Research Institute at the University of Southern California. ADNI data are disseminated by the Laboratory for Neuro Imaging at the University of Southern California.

## Conflict of Interest Statement

The authors declare that the research was conducted in the absence of any commercial or financial relationships that could be construed as a potential conflict of interest.

## Footnotes

1 adni.loni.usc.edu

2 http://surfer.nmr.mgh.harvard.edu/

## Abbreviations

CC: corpus callosum
CTH: cortical thickness
LLD: late-life depression
RF: random forest method
R-HC: right hippocampus
R-VD: right ventral diencephalon
SV: subcortical volumetric measurement
