# Supplementary material for: MRI-Based Classification Models in Prediction of Mild Cognitive Impairment and Dementia in Late-Life Depression
Source: Front Aging Neurosci. 2017 Feb 2;9:13. doi: 10.3389/fnagi.2017.00013 (PMC5288688; doi:10.3389/fnagi.2017.00013)
Supplement: Supplementary file 1 [file Table_1.DOCX]

**Table 1 MRI scanning parameters and number of participants**

| center N | Scanner | Slice thickness  (mm) | TR(ms) | TE(ms) | FA(°) | MFS  (Tesla) | CS | MCI-DEM | total |
| --- | --- | --- | --- | --- | --- | --- | --- | --- | --- |
| 1 | Symphony | 1.2 | 2400 | 3.71 | 8 | 1,5 | 3 | 3 | 6 |
| 2 | Avanto | 1.2 | 2400 | 3.79 | 8 | 1,5 | 3 | 1 | 4 |
| 3 | Avanto | 1.2 | 2400 | 3.61 | 8 | 1,5 | 9 | 5 | 11 |
| 4 | Symphony | 1.2 | 2400 | 2.88 | 8 | 1,5 | 2 | 0 | 2 |
| 5 | Avanto | 1.2 | 2400 | 3.61 | 8 | 1,5 | 14 | 12 | 26 |
| 6 | Philips Intera | 1.2 | 6.73 | 3.1 | 8 | 3 | 9 | 8 | 17 |
| Notes: TR-repetition time, TE-echo time, FA-flip angle, MFS - Magnetic field strength | | | | | | | | | |
