# Supplementary material for: MRI-Based Classification Models in Prediction of Mild Cognitive Impairment and Dementia in Late-Life Depression
Source: Front Aging Neurosci. 2017 Feb 2;9:13. doi: 10.3389/fnagi.2017.00013 (PMC5288688; doi:10.3389/fnagi.2017.00013)
Supplement: Supplementary file 2 [file Table_2.DOCX]

**Table 2**

| **Measurements used as input for the classification models** |
| --- |
| Cortical thicknesses |
| Inferior occipital gyrus and sulcus |
| Paracentral lobule and sulcus |
| Subcentral gyrus (central operculum) and sulci |
| Transverse frontopolar gyri and sulci |
| Anterior part of the cingulate gyrus and sulcus |
| Middle-anterior part of the cingulate gyrus and sulcus |
| Middle-posterior part of the cingulate gyrus and sulcus |
| Posterior-dorsal part of the cingulate gyrus |
| Posterior-ventral part of the cingulate gyrus |
| Cuneus |
| Opercular part of the inferior frontal gyrus |
| Orbital part of the inferior frontal gyrus |
| Triangular part of the inferior frontal gyrus |
| Middle frontal gyrus |
| Superior frontal gyrus |
| Long insular gyrus and central sulcus of the insula |
| Short insular gyri |
| Middle occipital gyrus |
| Superior occipital gyrus |
| Lateral occipito-temporal gyrus (fusiform gyrus) |
| Lingual gyrus, ligual part of the medial occipito-temporal gyrus |
| Parahippocampal gyrus, parahippocampal part of the medial occipito-temporal gyrus |
| Orbital gyri |
| Angular gyrus |
| Supramarginal gyrus |
| Superior parietal lobule |
| Postcentral gyrus |
| Precentral gyrus |
| Precuneus |
| Straight gyrus, Gyrus rectus |
| Subcallosal area, subcallosal gyrus |
| Anterior transverse temporal gyrus |
| Lateral aspect of the superior temporal gyrus |
| Planum polare of the superior temporal gyrus |
| Planum temporale or temporal plane of the superior temporal gyrus |
| Inferior temporal gyrus |
| Middle temporal gyrus |
| Horizontal ramus of the anterior segment of the lateral sulcus (fissure) |
| Vertical ramus of the anterior segment of the lateral sulcus (fissure) |
| Posterior ramus (or segment) of the lateral sulcus (fissure) |
| Occipital pole |
| Temporal pole |
| Calcarine sulcus |
| Central sulcus (Rolando’s fissure) |
| Marginal branch (or part) of the cingulate sulcus |
| Anterior segment of the circular sulcus of the insula |
| Inferior segment of the circular sulcus of the insula |
| Superior segment of the circular sulcus of the insula |
| Anterior transverse collateral sulcus |
| Posterior transverse collateral sulcus |
| Inferior frontal sulcus |
| Middle frontal sulcus |
| Superior frontal sulcus |
| Sulcus intermedius primus (of Jensen) |
| Intraparietal sulcus (interparietal sulcus) and transverse parietal sulci |
| Middle occipital sulcus and lunatus sulcus |
| Superior occipital sulcus and transverse occipital sulcus |
| Anterior occipital sulcus and preoccipital notch (temporo-occipital incisure) |
| Lateral occipito-temporal sulcus |
| Medial occipito-temporal sulcus (collateral sulcus) and lingual sulcus |
| Lateral orbital sulcus |
| Medial orbital sulcus (olfactory sulcus) |
| Orbital sulci (H-shaped sulci) |
| Parieto-occipital sulcus (or fissure) |
| Pericallosal sulcus (S of corpus callosum) |
| Postcentral sulcus |
| Inferior part of the precentral sulcus |
| Superior part of the precentral sulcus |
| Suborbital sulcus (sulcus rostrales, supraorbital sulcus) |
| Subparietal sulcus |
| Inferior temporal sulcus |
| Superior temporal sulcus (parallel sulcus) |
|  |
| Volumetric measures |
| Left Lateral Ventricle |
| Left Inferior Lateral Ventricle |
| Left Cerebellum White Matter |
| Left Cerebellum Cortex |
| Left Thalamus Proper |
| Left Caudate |
| Left Putamen |
| Left Pallidum |
| 3rd Ventricle |
| 4th Ventricle |
| Brain Stem |
| Left Hippocampus |
| Left Amygdala |
| Cerebrospinal Fluid volume |
| Left Accumbens area |
| Left Ventral Diencephalon |
| Right Lateral Ventricle |
| Right Inferior Lateral Ventricle |
| Right Cerebellum White Matter |
| Right Cerebellum Cortex |
| Right Thalamus Proper |
| Right Caudate |
| Right Putamen |
| Right Pallidum |
| Right Hippocampus |
| Right Amygdala |
| Right Accumbens area |
| Right Ventral Diencephalon |
| Terminal Ventricle |
| CC Posterior |
| CC Middle Posterior |
| CC Central |
| CC Middle Anterior |
| CC Anterior |
| Left Cortex Volume |
| Right Cortex Volume |
| Left Cortical White Matter Volume |
| Right Cortical White Matter Volume |
